# Comparing multimodal physiological responses to social and physical pain in healthy participants

**DOI:** 10.3389/fpubh.2024.1387056

**Published:** 2024-04-03

**Authors:** Eun-Hye Jang, Young-Ji Eum, Daesub Yoon, Jin-Hun Sohn, Sangwon Byun

**Affiliations:** ^1^Mobility User Experience Research Section, Electronics Telecommunication and Research Institute, Daejeon, Republic of Korea; ^2^Bio-Chemical Analysis Team, Korea Basic Science Institute, Cheongju, Republic of Korea; ^3^Department of Psychology, Chungnam National University, Daejeon, Republic of Korea; ^4^Department of Electronics Engineering, Incheon National University, Incheon, Republic of Korea

**Keywords:** social pain, physical pain, physiological signals, multimodal, autonomic nervous system

## Abstract

**Background:**

Previous physiology-driven pain studies focused on examining the presence or intensity of physical pain. However, people experience various types of pain, including social pain, which induces negative mood; emotional distress; and neural activities associated with physical pain. In particular, comparison of autonomic nervous system (ANS) responses between social and physical pain in healthy adults has not been well demonstrated.

**Methods:**

We explored the ANS responses induced by two types of pain—social pain, associated with a loss of social ties; and physical pain, caused by a pressure cuff—based on multimodal physiological signals. Seventy-three healthy individuals (46 women; mean age = 20.67 ± 3.27 years) participated. Behavioral responses were assessed to determine their sensitivity to pain stimuli. Electrocardiogram, electrodermal activity, photoplethysmogram, respiration, and finger temperature (FT) were measured, and 12 features were extracted from these signals.

**Results:**

Social pain induced increased heart rate (HR) and skin conductance (SC) and decreased blood volume pulse (BVP), pulse transit time (PTT), respiration rate (RR), and FT, suggesting a heterogeneous pattern of sympathetic–parasympathetic coactivation. Moreover, physical pain induced increased heart rate variability (HRV) and SC, decreased BVP and PTT, and resulted in no change in FT, indicating sympathetic-adrenal-medullary activation and peripheral vasoconstriction.

**Conclusion:**

These results suggest that changes in HR, HRV indices, RR, and FT can serve as markers for differentiating physiological responses to social and physical pain stimuli.

## 1 Introduction

Pain is not only a distressing experience in itself, but it can also have adverse effects on every aspect of life, including mood and the ability to perform daily roles (1). When pain persists and is not effectively managed, thus becoming chronic, it can cause emotional distress, impeding daily activities and affecting long-term health (2). In addition, pain is closely related to the quality of life (QOL) assessment, which reflects how satisfied and happy an individual’s life is (1, 3). Pain encompasses cognitive, motivational, emotional, behavioral, and physical components, with the emotional aspect having the most significant impact on the QOL (4). Therefore, pain needs to be appropriately addressed to avoid detrimental outcomes in physical and mental health conditions. To achieve this, quantitative pain assessment can play an important role in obtaining accurate information about the pain, such as intensity, duration, and type (5).

The quantitative evaluation of pain based on physiological signals has attracted attention because of its potential to address health problems related to pain (5). For example, it is vital to quantify pain in hospitalized patients who cannot talk, and an accurate assessment of pain can assist healthcare providers understand the severity of a patient’s condition and develop suitable treatments (6). Physiological signals are advantageous because signal acquisition by non-invasive sensors is comparatively straightforward, and physiological reactions to emotional stimuli exhibit considerable similarity across diverse societies and cultures (7). Previous physiologically-driven pain studies have demonstrated significant associations between several physiological signals and the presence of pain (5). Recently, automated methods for recognizing and analyzing pain have been demonstrated using machine-learning algorithms (8, 9).

Previous pain studies mainly focused on physical pain and estimated its intensity levels using various physiological signals, including electroencephalogram, electromyogram (EMG), electrodermal activity (EDA), electrocardiogram (ECG), and photoplethysmogram (PPG) (8–12). However, people experience various forms of pain including the termination of a social relationship as well as noxious bodily stimuli. Pain, by definition, is “an unpleasant sensory and emotional experience associated with actual or potential tissue damage or described in terms of such damage” (13). Pain is not only limited to physical pain but also includes a variety of negative emotional responses related to social injuries or perceptions of interpersonal rejection or loss (14).

Unlike physical pain—defined as “an unpleasant experience associated with actual or potential tissue damage or noxious physical stimuli”—social pain is “an unpleasant experience associated with actual or potential damage to one’s sense of social connection or value owing to social rejection, exclusion, negative social evaluation, or loss” (15). In personality psychology, social pain refers to “the activation of pain affect in response to threats to, or losses of, social connection” (16). Hurt feelings are a subtype of social pain (16). Panksepp (14) explained that hurt feelings derived from these experiences are key emotional markers of social pain. From a clinical perspective, both physical and social pain are important. When pain is prolonged without adequate intervention or therapy, it can evoke a sense of helplessness (17). In severe cases, psychological distress that results from social loss can precipitate depressive episodes and even evoke suicidal thoughts (18).

On the surface, the two types of pain seem quite distinct. However, most pain studies have noted that the pain associated with a relationship break-up or the loss of a loved one is similar to the pain experienced upon physical injury, and researchers endeavored to tackle this issue by investigating the neural overlap between the two types of pain (19). Physiological results suggest that social and physical pain function through overlapping mechanisms involving the anterior cingulate cortex (ACC), periaqueductal gray brain structures, and the opioid and oxytocin neuroendocrine systems (20). The ACC—an area associated with the negative feelings of physical pain—responds to social exclusion tests (21). Endogenous brain opioid systems—known to regulate distress caused by physical pain—are neurochemical regulators of distress associated with social pain (15). Consistently, studies that investigated diverse scenarios capable of inducing social pain, such as social exclusion or bereavement, found activity in multiple neural regions that are linked to physical pain (15, 19). However, the distinctions between alterations of physiological signals induced by social and physical pain have not been thoroughly examined.

Previous studies on social pain have evaluated physiological responses but have mostly used negative evaluation tasks to induce pain (15). Although social pain also includes experiences in which a relationship is lost (15), physiological responses to social pain owing to the loss of a relationship require elucidation. Further, heart rate (HR) and blood pressure were primarily measured in previous studies on social pain (21, 22). Although these two features can represent changes in physiological responses, relying on these two measurements may not be sufficient to discriminate the activation patterns of the sympathetic nervous system (SNS) and parasympathetic nervous system (PNS), which is important for understanding the dysregulation of physiological systems caused by social pain.

In recent research on physiological sensing to evaluate acute pain, some studies have utilized a combination of multiple physiological signals as potential indicators for pain recognition, most commonly evaluated through EDA, ECG, and PPG (23). Additionally, skin temperature and respiratory features have been used as metrics to evaluate pain. For example, local skin temperature (e.g., fingertip) decreases after experiencing painful stimuli (12, 24); a decrease in respiration rate (RR), which measures the number of breaths per minute, has been reported in response to pain (24, 25). Since each of these physiological measures has its constraints and advantages, integrating them can improve pain assessment.

Our aim was to explore the changes in autonomic nervous system (ANS) responses induced by two types of pain—social pain associated with a loss of social ties and physical pain caused by a pressure cuff—using multimodal physiological signals. We obtained ECG, EDA, PPG, respiration (RESP), and finger temperature (FT) data and then extracted the physiological features that evaluate the ANS response from the measured signals. These features were compared between social and physical pain to investigate whether there were differences in the ANS responses between pain types. We hypothesized that social and physical pain stimuli induce ANS responses reflected by multimodal signals, owing to the critical role of the ANS in response to mental and physical stress (26). In addition, we hypothesized that the SNS and PNS activity induced by the two pain types would show distinct patterns. The main contribution of this study is that we compared social and physical pain based on multimodal signals instead of focusing on the presence or absence of pain or pain intensity using fewer features, which provides new insights for understanding the difference in physiological responses to social and physical pain.

## 2 Materials and methods

### 2.1 Participants

Seventy-three healthy individuals (46 women; mean age = 20.67 ± 3.27 years) participated. No one reported a history of medical illness; use of neurological or psychiatric medication; or use of medication that could affect the cardiovascular, respiratory, or central nervous systems. All participants were introduced to the experimental procedure and signed informed consent forms before study commencement. This study was approved by the Institutional Review Board of Chungnam National University (no. 201309-SB-041-01). All participants received $30 USD.

### 2.2 Emotion-provoking stimuli

**Figure 1:**
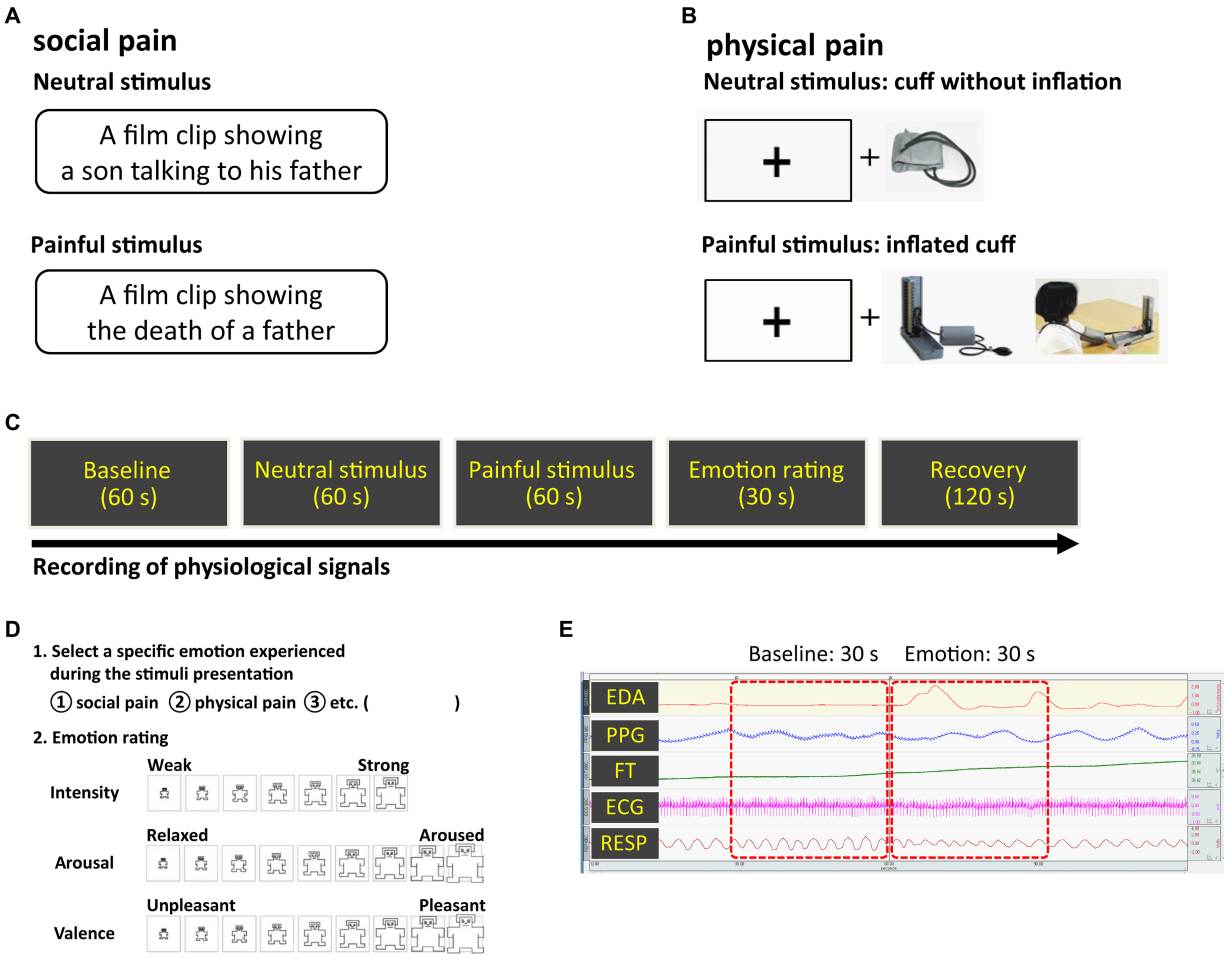
Experimental methods **(A)** social pain stimulus **(B)** physical pain stimulus **(C)** overall procedure **(D)** emotion rating scale **(E)** physiological signals observed during the experiment. Pain-inducing sessions were presented in random order.

We specified social pain as the loss of a loved one to death, which could cause tremendous pain owing to the loss of access to a particular relational partner (27) and physical pain as a physical sensation that causes discomfort. To induce social pain, we used a 60-s long film clip showing a son grieving over his father’s death, which was excerpted from a Korean drama entitled *Ruler of Your Own World* (28). We selected an audiovisual film clip as the emotion-provoking stimulus because film stimuli are readily standardized, dynamic rather than static, involve no deception, and demonstrate a considerable level of ecological validity (29). A 60-s long neutral film clip was excerpted from the same drama depicting a son talking to his father. To induce physical pain, a conventional blood pressure cuff was placed on the participant’s non-dominant arm and progressively inflated to a peak pressure of 250 mmHg. Cuff inflation took 60 s, including maintaining the maximum pressure for 5 s. Simultaneously, during cuff inflation, participants were required to look at a plus sign (+) presented on the monitor. The plus sign was displayed in black on a white background to minimize the impact of color perception on the subject’s response to the stimulus. As a neutral physical stimulus, the cuff was applied to the arm for 60 s without any inflation, and participants looked at the plus sign on the monitor. The stimuli were counterbalanced to minimize the effects of order and intensity. The pain-inducing stimuli are shown in Figure 1.

### 2.3 Procedure

Prior to the experiment, participants had a period of adaptation to feel comfortable in the laboratory setting. Electrodes for acquiring physiological signals were attached to the wrists, fingers, ankles, and chest. The following steps were applied to both social and physical pain: during the 60-s long baseline measurement of physiological signals, participants were asked to rest. Neutral and pain-inducing stimuli were then presented to the participants as described above, both of which lasted for 60 s. After the stimuli presentation, participants were asked to select the specific emotions they experienced during exposure (i.e., social pain, physical pain), and rate their dimensions (i.e., intensity, valence, and arousal) (Figure 1). Valence and arousal were rated on a scale ranging from −4 (negative valence and low arousal, respectively) to 4 (positive valence and high arousal, respectively). Intensity was rated on a seven-point Likert scale: 1 (“least”) to 7 (“most”). The rating duration was 30 s. After the ratings, they were given 2 min to be debriefed and recover from their emotional states.

### 2.4 Physiological signal recordings

ECG, EDA, FT, RESP, and PPG signals were measured and analyzed using MP100WS and AcqKnowledge software (version 3.7.1) from Biopac Systems Inc. (Goleta, CA, United States). For all channels, the sampling frequency was set to 250 Hz. Amplification and bandpass filtering were applied to the collected signals. The ECG electrodes were attached to both wrists and the left ankle based on the lead-I configuration. The EDA signal was acquired using AgCl electrodes of 8-mm diameter attached to the volar surface of the distal phalanges of the index and middle fingers of the non-dominant hand. The electrodes were filled with a 0.05 molar isotonic NaCl paste to ensure a stable connection between the electrodes and the skin. The PPG sensor was placed on the first joint of the non-dominant thumb. The FT electrode was attached to the first joint of the non-dominant ring finger. The RESP sensor was wrapped around the chest using a Velcro strap to measure the expansion and contraction of the chest cavity based on the Hall effect. We chose ECG, EDA, FT, RESP, and PPG signals because they have been widely studied as important physiological signals that reflect ANS activity. Although these signals can be affected by motion or other environmental factors, their measurement using non-invasive sensors is simple and allows for real-time recording of a user’s state. These physiological signals are less influenced by social and cultural differences and are resilient against social masking or factitious emotional expressions because they can be captured through spontaneous emotional responses (30).

### 2.5 Feature extraction

Data from the last 30 s of the baseline and the first 30 s of the neutral and pain-inducing states were used in the analysis (Figure 1). Twelve features were extracted from each experimental state: baseline, neutral, and pain-induced states (Table 1). Therefore, 36 features were extracted for each emotion type. HR, standard deviation of NN intervals (SDNN), root mean square of successive differences between adjacent NNs (RMSSD), and percentage of successive NNs that differed by more than 50 ms (pNN50) were extracted as the time-domain features of HR variability (HRV) from the ECG signals. Normalized powers in low frequency band (LFnu, 0.04–0.15 Hz) and high frequency band (HFnu, 0.15–0.4 Hz), and the LF/HF ratio were evaluated from HRV frequency spectral analysis (31). HFnu is the ratio of HF power to the sum of HF and LF power. LFnu is the ratio of LF power to the sum of HF and LF power. Therefore, LFnu and HFnu always add up to 1. LF/HF ratio is the ratio of LFnu to HFnu. Since LFnu and HFnu were exchangeable with a perfect linear association, we only used HFnu and LF/HF ratio in this study (32). Skin conductance level (SCL) and response (SCR) amplitude were analyzed as EDA indicators as they represent changes in the electrical properties of the skin that are attributable to the functioning of sweat glands and are interpreted as conductance (33). SCL was the average of the tonic component of EDA. SCRs represent the phasic waves of the EDA signal. In the current study, SCR was calculated by averaging the SCR amplitude (0.05 μs or greater) of all the specific SCR events for a 30-s interval (34). The blood volume pulse (BVP) was calculated by averaging the BVP range, which refers to the difference between the highest and lowest values of each pulse wave, over a 30-s interval (35). Pulse transit time (PTT) was extracted from the ECG and PPG signals as it is a measure of the elapsed time between the R-peak of the ECG and the arrival of the pulse wave at the finger. RR was calculated by counting the number of breaths, defined as the number of times the chest increased during the baseline and emotional states. The FT was calculated by averaging the FT values for the 30-s interval.

**Table 1:** Description of the physiological features used in the current study.

Signals	Features	Definition
ECG	HR (beat/min)	Average of HR
	SDNN (ms)	Standard deviation of NN intervals
	RMSSD (ms)	Root mean square of successive differences between adjacent NNs
	pNN50 (%)	Percentage of successive NNs that differed by more than 50 ms
	HFnu	HFnu = HF power / (LF power + HF power), where LF power and HF power are the absolute powers in the LF (0.04–0.15 Hz) and HF (0.15–0.4 Hz) bands, respectively.
	LF/HF	Ratio of LFnu to HFnu
RESP	RR (breath/min)	Number of breaths per minute
PPG	BVP (V)	Average of BVP range
ECG, PPG	PTT (ms)	Elapsed time between the R-peak of the ECG and the arrival of the pulse wave at the finger
EDA	SCL (μS)	Tonic level of electrical conductivity of skin
	SCR (μS)	Average of the SCR amplitude (0.05 μS or greater) of all the specific SCR events
Temperature	FT (°C)	Average of FT

In this study, we utilized the predominant method for measuring and analyzing emotion-specific ANS responses. Specifically, most previous studies averaged over 30- or 60-s intervals, while others frequently used interval durations of 0.5, 10, 120, 180, or 300 s (7). However, it has been observed that the average duration does not significantly influence the reported pattern of physiological responses (7), suggesting that a 30-s interval can be considered an appropriate ANS response measurement.

### 2.6 Statistical analysis

A paired t-test was used to compare the intensity ratings between two pain stimuli and to test differences in baseline feature values between two pain types. A two-way repeated-measures analysis of variance (ANOVA) was used to test the effects of the type of pain stimulus (social and physical) and experimental state (baseline, neutral, and painful) on physiological features. As a post-hoc analysis, between-pain type and between-state estimated marginal means were compared with confidence interval adjustments using the least significant difference method. The Bonferroni correction was used for multiple pairwise comparisons among experimental states for each pain type (*p* < 0.0167). All statistical analyses were performed using SPSS (version 21.0; IBM, Armonk, NY, United States).

## 3 Results

### 3.1 Participants’ rating on the dimensions of emotion

**Figure 2:**
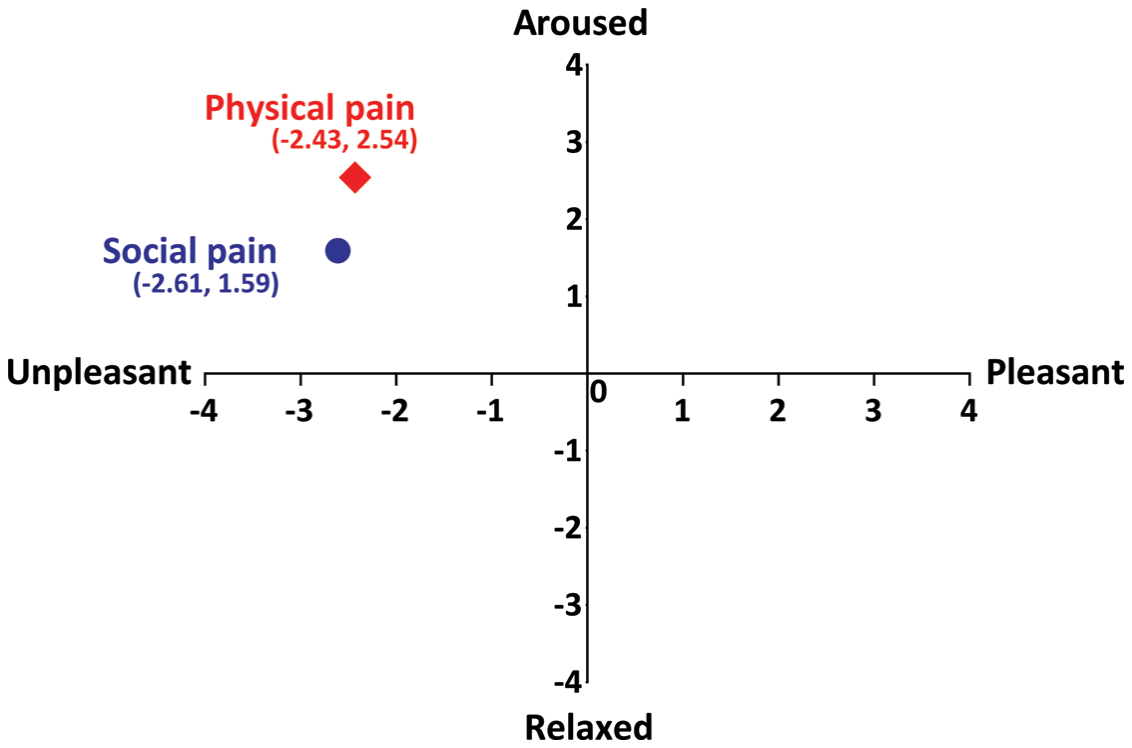
Participants’ ratings of social and physical pain on the dimension of emotion. Vertical axis represents the arousal and horizontal axis represents the valence.

Participants’ ratings of the physical-and social pain-inducing stimuli on the dimensions of emotion (arousal and valence) showed that both stimuli were located in the same quadrant (Figure 2). The mean intensities of social and physical pain-inducing stimuli were 5.74 ± 0.71 and 5.63 ± 0.72, respectively. No significant differences were found in the intensity ratings between two pain stimuli (*t*(72) = 0.599, *p* = 0.551).

### 3.2 Physiological responses induced by social and physical pain

Table 2 shows the mean values of the physiological features representing the ANS responses during the three experimental states (baseline, neutral, and painful) for both types of pain stimuli. No significant differences were observed in the baseline feature values between two pain types.

**Table 2:** Mean values (± SD) of physiological features measured during three experimental states in social and physical pain stimuli.

Feature	Social pain			Physical pain			*t* (*p*-value)^a^
	Baseline	Neutral	Painful	Baseline	Neutral	Painful	
HR (beat/min)	79.64 ± 3.94	80.11 ± 4.18	82.37 ± 6.08	79.60 ± 4.06	77.07 ± 5.91	79.74 ± 6.33	0.083 (0.934)
SDNN (ms)	34.88 ± 6.58	31.99 ± 6.29	37.68 ± 8.65	34.30 ± 6.66	34.49 ± 7.60	40.32 ± 9.66	0.337 (0.737)
RMSSD (ms)	28.67 ± 6.09	27.67 ± 5.50	29.40 ± 6.78	28.59 ± 5.75	30.63 ± 6.84	33.20 ± 6.78	0.076 (0.939)
pNN50 (%)	11.48 ± 7.45	9.56 ± 6.15	11.55 ± 7.04	10.14 ± 6.26	12.97 ± 8.04	14.57 ± 6.69	1.071 (0.288)
HFnu	0.59 ± 0.07	0.62 ± 0.07	0.62 ± 0.06	0.57 ± 0.08	0.58 ± 0.07	0.54 ± 0.06	1.202 (0.233)
LF/HF	0.82 ± 0.27	0.72 ± 0.25	0.67 ± 0.17	0.97 ± 0.36	0.87 ± 0.32	0.99 ± 0.26	−1.797 (0.077)
RR (breath/min)	3.83 ± 0.59	3.55 ± 0.38	3.45 ± 0.45	3.82 ± 0.57	3.66 ± 0.43	3.69 ± 0.51	0.100 (0.920)
BVP (V)	0.16 ± 0.05	0.14 ± 0.05	0.09 ± 0.03	0.17 ± 0.05	0.17 ± 0.05	0.08 ± 0.03	−0.408 (0.685)
PTT (ms)	275.45 ± 10.55	273.30 ± 10.21	266.62 ± 13.57	274.64 ± 9.79	276.53 ± 12.80	268.57 ± 12.84	0.593 (0.555)
SCL (μS)	0.22 ± 0.07	0.24 ± 0.76	0.30 ± 0.09	0.22 ± 0.07	0.23 ± 0.07	0.32 ± 0.08	0.890 (0.376)
SCR (μS)	0.07 ± 0.16	0.20 ± 0.25	0.62 ± 0.36	0.08 ± 0.10	0.08 ± 0.09	0.91 ± 0.35	−0.338 (0.736)
FT (°C)	32.24 ± 1.15	32.15 ± 1.20	32.01 ± 1.08	32.07 ± 1.24	31.90 ± 1.29	31.91 ± 1.28	1.154 (0.262)

a Differences in baseline values between two pain types.

#### 3.2.1 Physiological features

Table 3 shows the results of the two-way repeated-measures ANOVA, which was conducted to analyze the effects of the type of pain stimulus and experimental state on each physiological feature, using a 2 (pain type: social and physical) × 3 (experimental state: baseline, neutral, and painful) within-participant model.

**Table 3:** Results from a two-way repeated-measures analysis of variance on the effects of the pain type and the experimental state on individual physiological features.

		SS	df	MS	*F*	*p*	*η* ^2^
HR	Pain type	397.09	1	397.09	12.58	**0.001**	0.149
	State	445.78	2	222.89	5.34	**0.006**	0.069
	Interaction	193.22	2	96.61	2.30	0.104	0.031
SDNN	Pain type	252.88	1	252.88	1.47	0.230	0.020
	State	269.29	2	1324.65	8.96	**< 0.001**	0.111
	Interaction	240.34	2	120.17	1.08	0.344	0.015
RMSSD	Pain type	543.38	1	543.38	14.93	**< 0.001**	0.172
	State	584.32	2	292.16	7.05	**0.001**	0.089
	Interaction	303.27	2	151.63	3.12	**0.047**	0.042
pNN50	Pain type	314.23	1	314.23	7.35	**0.008**	0.093
	State	413.99	2	206.99	3.96	**0.021**	0.052
	Interaction	506.86	2	253.43	3.97	**0.021**	0.052
HFnu	Pain type	0.28	1	0.28	18.96	**< 0.001**	0.208
	State	0.06	2	0.03	2.03	0.135	0.027
	Interaction	0.08	2	0.04	2.85	0.061	0.038
LF/HF	Pain type	4.72	1	4.72	24.17	**< 0.001**	0.251
	State	0.76	2	0.38	1.59	0.206	0.022
	Interaction	0.79	2	0.39	1.95	0.146	0.026
RR	Pain type	1.43	1	1.43	3.38	0.070	0.045
	State	5.54	2	2.77	3.35	**0.038**	0.045
	Interaction	1.20	2	0.60	1.35	0.264	0.018
BVP	Pain type	0.00	1	0.00	0.92	0.341	0.013
	State	0.57	2	0.28	55.88	**< 0.001**	0.437
	Interaction	0.02	2	0.01	2.85	0.061	0.038
PTT	Pain type	233.14	1	233.14	2.61	0.111	0.035
	State	5309.85	2	5309.85	23.04	**< 0.001**	0.242
	Interaction	311.18	2	155.59	1.87	0.157	0.025
SCL	Pain type	0.00	1	0.00	0.05	0.826	0.001
	State	0.66	2	0.33	61.67	**< 0.001**	0.461
	Interaction	0.02	2	0.01	1.40	0.250	0.019
SCR	Pain type	0.40	1	0.40	2.75	0.101	0.037
	State	43.15	2	21.57	91.44	**< 0.001**	0.559
	Interaction	3.24	2	1.62	9.31	**< 0.001**	0.114
FT	Pain type	3.94	1	3.94	5.89	**0.018**	0.076
	State	2.29	2	1.15	3.59	**0.030**	0.048
	Interaction	0.18	2	0.09	0.16	0.855	0.002

Bold = significant results.

##### 3.2.1.1 HR

There were significant main effects of pain type (*F*(1, 72) = 12.580, *p* < 0.001, partial *η*^2^ = 0.149) and experimental state (*F*(2, 144) = 5.343, *p* = 0.006, partial *η*^2^ = 0.069) on HR. There was no interaction between the pain type and experimental state (*F*(2, 144) = 2.304, *p* = 0.104, partial *η*^2^ = 0.031). HR in social pain (80.71 ± 1.00) was higher than in physical pain (78.80 ± 0.89, corrected *p* = 0.001). Post-hoc analysis revealed that HR in painful state (81.05 ± 1.14) was significantly higher than in baseline (79.62 ± 0.90, corrected *p* = 0.040) and neutral states (78.59 ± 0.97, corrected *p* = 0.007).

##### 3.2.1.2 SDNN

The main effect of pain type was non-significant (*F*(1, 72) = 1.466, *p* = 0.230, partial *η*^2^ = 0.020); however, the experimental state showed a significant main effect on SDNN (*F*(2, 144) = 8.961, *p* < 0.001, partial *η*^2^ = 0.111). There was no significant interaction between the pain type and experimental state (*F*(2, 144) = 1.076, *p* = 0.344, partial *η*^2^ = 0.015). Post-hoc analysis indicated that SDNN in painful state (39.00 ± 1.79) was significantly higher than in baseline (34.59 ± 1.29, corrected *p* = 0.009) and neutral states (33.24 ± 1.44, corrected *p* < 0.001).

##### 3.2.1.3 RMSSD

There were main effects of pain type (*F*(1, 72) = 14.934, *p* < 0.001, partial *η*^2^ = 0.172) and experimental state (*F*(2, 144) = 7.051, *p* = 0.001, partial *η*^2^ = 0.089) on RMSSD. There was an interaction between the pain type and experimental state (*F*(2, 144) = 3.119, *p* = 0.047, partial *η*^2^ = 0.042). RMSSD in social pain (28.58 ± 1.31) was lower than in physical pain (30.81 ± 1.35, corrected *p* < 0.001). Post-hoc analysis indicated that RMSSD in painful state (31.30 ± 1.47) was higher than in baseline (28.63 ± 1.30, corrected *p* = 0.003) and neutral states (29.15 ± 1.35, corrected *p* = 0.011).

##### 3.2.1.4 pNN50

There were main effects of pain type (*F*(1, 72) = 7.350, *p* = 0.008, partial *η*^2^ = 0.093) and experimental state (*F*(2, 144) = 3.961, *p* = 0.021, partial *η*^2^ = 0.052) on pNN50. There was an interaction between pain type and experimental state (*F*(2, 144) = 3.968, *p* = 0.021, partial *η*^2^ = 0.052). pNN50 in social pain (10.86 ± 1.44) was significantly lower than in physical pain (12.56 ± 1.48, corrected *p* = 0.008). Post-hoc analysis indicated that pNN50 in painful state (13.06 ± 1.50) was significantly higher than in baseline (10.81 ± 1.49, corrected *p* = 0.020).

##### 3.2.1.5 HFnu

There was a main effect of pain type on HFnu (*F*(1, 72) = 18.962, *p* < 0.001, partial *η*^2^ = 0.208) but not in experimental state (*F*(2, 144) = 2.029, *p* = 0.135, partial *η*^2^ = 0.027). There was no interaction between pain type and experimental state (*F*(2, 144) = 2.847, *p* = 0.061, partial *η*^2^ = 0.038).

##### 3.2.1.6 LF/HF

There was a main effect of pain type on LF/HF (*F*(1, 72) = 24.171, *p* < 0.001, partial *η*^2^ = 0.251) but not in experimental state (*F*(2, 144) = 1.597, *p* = 0.206, partial *η*^2^ = 0.022). There was no interaction between pain type and experimental state (*F*(2, 144) = 1.949, *p* = 0.146, partial *η*^2^ = 0.026).

##### 3.2.1.7 RR

The main effect of pain type was non-significant (*F*(1, 72) = 3.379, *p* = 0.070, partial *η*^2^ = 0.045). Experimental state had a main effect on RR (*F*(2, 144) = 3.354, *p* = 0.038, partial *η*^2^ = 0.045). There was no significant interaction between pain type and experimental state (*F*(2, 144) = 1.345, *p* = 0.264, partial *η*^2^ = 0.018). Post-hoc analysis indicated that RR in baseline (3.83 ± 0.13) was higher than in neutral (3.61 ± 0.09, corrected *p* = 0.015) or painful (3.57 ± 0.09, corrected *p* = 0.046) states.

##### 3.2.1.8 BVP

The main effect of pain type was non-significant (*F*(1, 72) = 0.919, *p* = 0.341, partial *η*^2^ = 0.013). There was a main effect of experimental state on BVP (*F*(2, 144) = 55.884, *p* < 0.001, partial *η*^2^ = 0.437). There was no interaction between pain type and experimental state (*F*(2, 144) = 2.851, *p* = 0.061, partial *η*^2^ = 0.038). Post-hoc analysis showed that BVP in painful state (0.09 ± 0.00) was lower than in baseline (0.17 ± 0.01, corrected *p* < 0.001) or neutral (0.16 ± 0.01, corrected *p* < 0.001) states.

##### 3.2.1.9 PTT

The main effect of pain type was non-significant (*F*(1, 72) = 2.606, *p* = 0.111, partial *η*^2^ = 0.035). There was a main effect of experimental state on PTT (*F*(2, 144) = 23.035, *p* < 0.001, partial *η*^2^ = 0.242). There was no interaction between pain type and experimental state (*F*(2, 144) = 1.873, *p* = 0.157, partial *η*^2^ = 0.025). Post-hoc analysis showed that the PTT in the painful state (267.60 ± 2.96) was lower than that in baseline (275.05 ± 2.29, corrected *p* < 0.001) or neutral (274.92 ± 2.49, corrected *p* < 0.001) states.

##### 3.2.1.10 SCL

The main effect of pain type was non-significant (*F*(1, 72) = 0.049, *p* = 0.826, partial *η*^2^ = 0.001). There was a main effect of experimental state on SCL (*F*(2, 144) = 61.671, *p* < 0.001, partial *η*^2^ = 0.461) but no interaction between the pain type and experimental state (*F*(2, 144) = 1.400, *p* = 0.250, partial *η*^2^ = 0.019). Post-hoc analysis indicated that SCL in painful state (0.31 ± 0.02) was higher than in baseline (0.22 ± 0.02, corrected *p < 0*.001) or neutral (0.23 ± 0.02, corrected *p* < 0.001) states.

##### 3.2.1.11 SCR

The main effect of pain type was non-significant (*F*(1, 72) = 2.752, *p* = 0.101, partial *η*^2^ = 0.037). There was a main effect of experimental state on SCR (*F*(2, 144) = 91.438, *p* < 0.001, partial *η*^2^ = 0.559). There was an interaction between pain type and experimental state (*F*(2, 144) = 9.305, *p* < 0.001, partial *η*^2^ = 0.114). Post-hoc analysis revealed that SCR in painful state (0.77 ± 0.07) was higher than in baseline (0.07 ± 0.02, corrected *p* < 0.001) or neutral (0.14 ± 0.04, corrected *p* < 0.001) states.

##### 3.2.1.12 FT

There were main effects of pain type (*F*(1, 72) = 5.899, *p* = 0.018, partial *η*^2^ = 0.076) and experimental state (*F*(2, 144) = 3.599, *p* = 0.030, partial *η*^2^ = 0.048) on FT; however, there was no interaction between pain type and experimental state (*F*(2, 144) = 0.157, *p* = 0.855, partial *η*^2^ = 0.002). FT in social pain (32.15 ± 0.26) was significantly higher than in physical pain (31.96 ± 0.29, corrected *p* = 0.018). Post-hoc analysis revealed that FT in baseline (32.16 ± 0.27) was higher than in neutral (32.03 ± 0.29, corrected *p* = 0.027) and painful (31.99 ± 0.27, corrected *p* = 0.013) states.

#### 3.2.2 Pairwise comparisons

**Figure 3:**
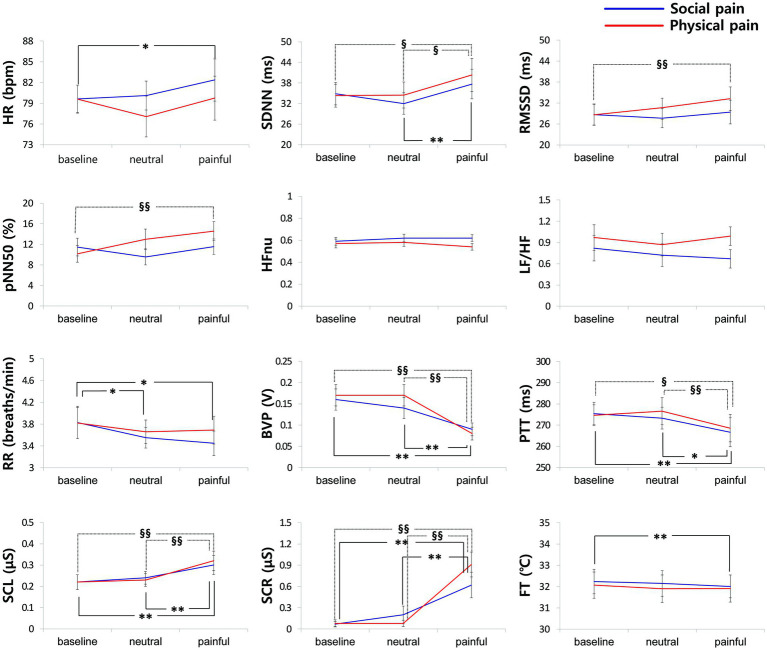
Physiological responses observed during the experimental states for each pain type. Significantly different pairwise comparisons among experimental states are indicated by asterisks for social pain (*p < 0.0167, **p < 0.001) and section signs for physical pain (§*p* < 0.0167, §§*p* < 0.001).

Figure 3 shows the physiological responses observed during the experimental states and indicates significantly different pairwise comparisons among the experimental states for each pain type, which were evaluated using post-hoc analyses. Table 4 lists the significantly different pairwise comparisons. HR showed significant differences between the painful state and baseline only in social pain. In the SDNN, there were significant differences between the neutral and painful states in both pain types. The RMSSD and pNN50 showed significant differences between the baseline and painful states in physical pain. The RR in the painful state was significantly lower than that in baseline for social pain. BVP and PTT in the painful state were significantly lower than those in the baseline and neutral states for both types of pain. There were significant increases in SCL and SCR during painful states compared with the baseline and neutral states for both types of pain. The FT in the painful state was significantly lower than that in baseline for social pain.

**Table 4:** Significantly different pairwise comparisons among experimental states for each pain type (Bonferroni correction for multiple comparisons, *p* < 0.0167).

	Social pain	Physical pain
HR	Pain > Baseline (*p* = 0.012)	N/A
SDNN	Pain > Neutral (*p* < 0.001)	Pain > Neutral (*p* = 0.015) Pain > Baseline (*p* = 0.011)
RMSSD	N/A	Pain > Baseline (*p* < 0.001)
pNN50	N/A	Pain > Baseline (*p* < 0.001)
HFnu	N/A	N/A
LF/HF	N/A	N/A
RR	Pain < Baseline (*p* = 0.008) Neutral < Baseline (*p* = 0.015)	N/A
BVP	Pain < Neutral (*p* < 0.001) Pain < Baseline (*p* < 0.001)	Pain < Neutral (*p* < 0.001) Pain < Baseline (*p* < 0.001)
PTT	Pain < Neutral (*p* = 0.001) Pain < Baseline (*p* < 0.001)	Pain < Neutral (*p* < 0.001) Pain < Baseline (*p* = 0.001)
SCL	Pain > Neutral (*p* < 0.001) Pain > Baseline (*p* < 0.001)	Pain > Neutral (*p* < 0.001) Pain > Baseline (*p* < 0.001)
SCR	Pain > Neutral (*p* < 0.001) Pain > Baseline (*p* < 0.001)	Pain > Neutral (*p* < 0.001) Pain > Baseline (*p* < 0.001)
FT	Pain < Baseline (*p* < 0.001)	N/A

N/A, not applicable.

## 4 Discussion

**Figure 4:**
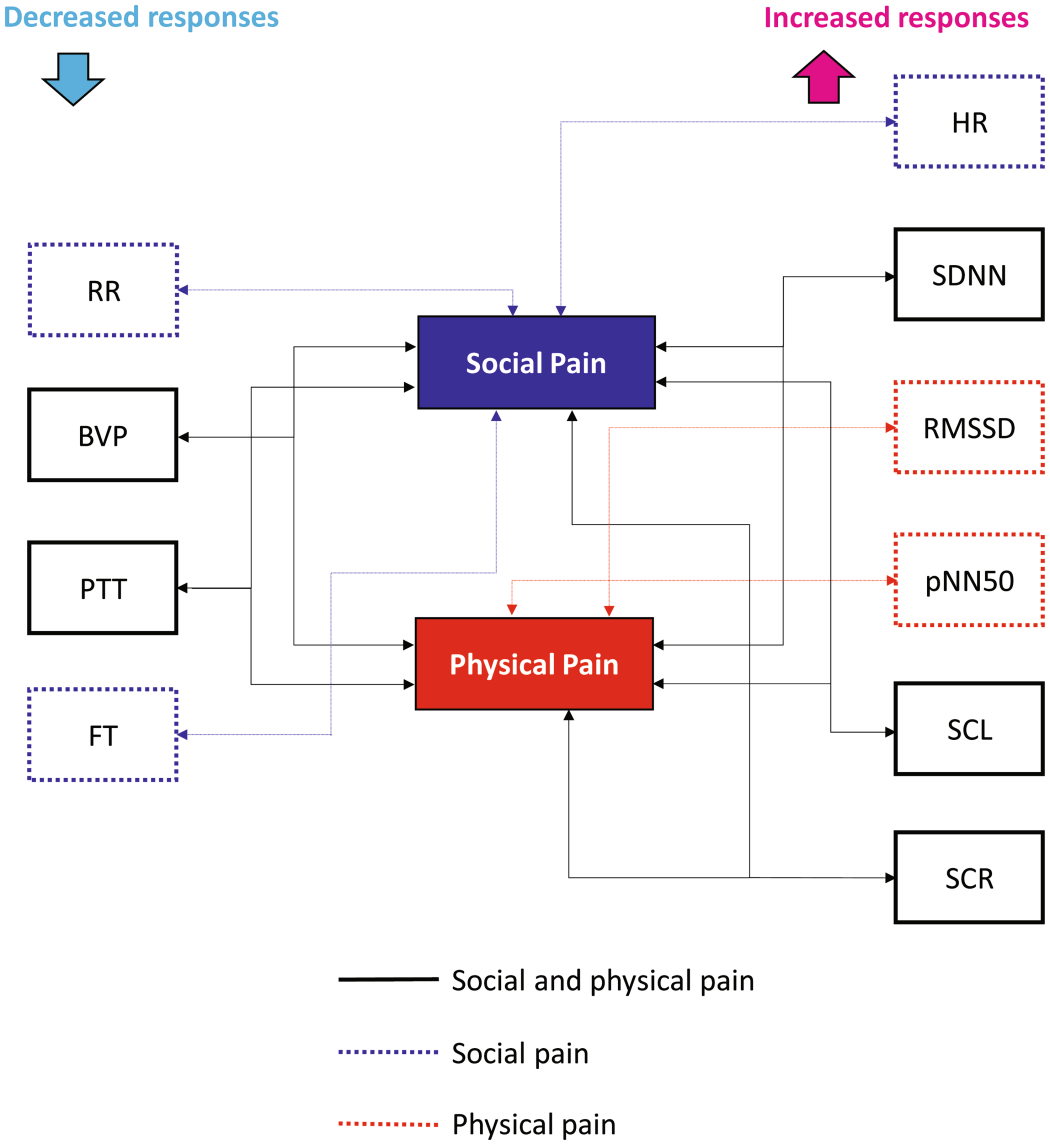
Physiological features that significantly responded to social or physical pain stimuli.

We identified changes in ANS responses induced by social and physical pain stimuli and compared the differences in their responses between the two pain types. Figure 4 shows the physiological features that significantly responded to social or physical pain stimuli. The following features showed similar ANS responses to both social and physical pain stimuli: increased SDNN, SCL, and SCR but decreased BVP and PTT during the painful state. Because the SDNN reflects the total variability in the NN intervals during the recording period, it increases when the HRV is large and irregular (36). Mikuckas et al. (37) showed that the SDNN is increased by mental stress caused by irritating music. SC activity measures the psychogalvanic reflex response, in which the SNS activation stimulates the palmar and plantar eccrine sweat glands in reaction to emotional arousal, such as stress and fear (38). With sympathetic activation, sweat is secreted onto the skin surface, resulting in increased EDA; that is, a measurable increase in SC (38). Therefore, increased SCR indicates sweat secretion owing to the activated SNS. SCR is related to SAM activation, which indicates pain progression (12). BVP is a metric that indicates the volume of blood flowing through vessels such as those in the finger. It also serves as an indicator of changes in the vascular bed that are caused by vasoconstriction or vasodilation and changes in the elasticity of the vascular walls, which indicate changes in blood pressure (39). A reduction in BVP from baseline in response to a stimulus suggests peripheral vasoconstriction in the finger and is linked to arousal caused by social pain (16). PTT is the duration from the occurrence of the R-peak in the ECG to the moment the pulse wave reaches the finger (40). It is influenced by changes in the heart’s contractile strength and the average arterial blood pressure. An increased PTT indicates suppression of SNS activation; thus, a significant decrease in PTT during the pain stimulus suggests sympathetic activation. In sum, pain-specific ANS responses to both pain stimuli are associated with the SAM and sympathetic activation of peripheral vasoconstrictions.

Contrastingly, the HR, RMSSD, pNN50, RR, and FT groups exhibited different responses to the pain stimuli. Social pain led to a significant increase in HR and a significant decrease in RR, and FT. It did non-significantly change the RMSSD or pNN50. However, physical pain induced a significant increase in RMSSD and pNN50, but did not affect HR, RR, or FT. HR is under the joint control of the SNS and PNS, and ample studies explored how the brain regulates the ANS in this process (41). The two branches of the ANS have opposite effects on the HR, with different response latencies. The SNS increases HR and blood pressure by enhancing adrenergic activity, whereas the PNS decreases HR through cholinergic activity, primarily targeting the sinoatrial node (26). It takes several seconds for the sympathetic system to increase the HR, but the PNS can affect the immediate subsequent heartbeat. Relative increases in SNS and PNS activities are associated with increases and decreases in HR, respectively (42). Thus, the increased HR caused by social pain suggests that activation of SNS mediated by adrenergic activity.

HRV features represent the autonomic regulation of the HR and can serve as an objective index of emotionality (43). RMSSD represents short-term variations in HR and is mostly affected by parasympathetic activity (44). It is correlated with HF power and indicates self-regulatory capacity (39). Similar to the RMSSD, pNN50 mostly reflects the effect of parasympathetic activity. Thus, RMSSD and pNN50 can monitor vagally mediated changes in HRV (29). Prior studies demonstrated an increase in the RMSSD using acute thermal pain, such as in the cold-pressor task (45) and hot immersion tests (46). Konstantinou et al. (47) showed that pNN50 was increased by a cold-pressor task when measured with a wearable device.

In this study, HFnu and LF/HF were not significantly affected by the pain stimuli; however, some previous studies demonstrated that pain induced significant changes in the HRV frequency domain features. LF/HF is calculated as a ratio between low frequency (LF, 0.04–0.15 Hz) power—influenced by both parasympathetic and sympathetic activities and high frequency (HF, 0.15–0.4 Hz) power—which reflects dominantly parasympathetic (vagal) activity (48). Stress, pain, and mental frustration are associated with an elevated LF/HF caused by either a relatively higher LF or lower HF (49). For example, an elevated LF reflects an increase in sympathetic baroreflex activity, which can be induced by painful stimuli, such as heat pain caused by contact thermodes (50). A decrease in the HF induced by painful stimulation has been reported, reflecting reduced vagal-parasympathetic activity (42). Physical pain induced by pressure leads to a significant increase in LF and LF/HF and a decrease in HF, indicating increased sympathetic activity (51). Contrastingly, a decrease in the LF/HF during periods of mental stress may be associated with improved modulation of emotional expression (52) and defensive reactions (53). However, HFnu and LF/HF were not as relevant as the other HRV features for differentiating between social and physical pain in our results.

RR is one of the most widely used respiratory indices, along with the respiratory period, respiratory depth, tidal volume, duty cycle, and respiratory variability (7). Physical pain induces an increase in respiratory frequency, flow, and volume (54). For example, RR was increased in healthy participants by tourniquet pain in the calf (55) and by saline infusion into the masseter muscle (56). In clinical studies, patients experiencing pain exhibited increased RR (57, 58), suggesting the potential of RR as an indicator of pain in severely ill adults and intensive care patients. These findings support the idea that hyperventilation acts as a respiratory stress response in situations involving uncontrollable stress, fear, and pain (59, 60). However, we did not observe a significant change in RR with a physical pain stimulus. In addition, RR can be significantly affected by emotional changes. For example, the arousal caused by negative emotions induces shallower and more rapid breathing (61, 62), which could result in decreased blood carbon dioxide (7). Our results showed a significant decrease in RR during social pain, which may be related to deactivation of the sadness response—characterized by sympathetic withdrawal (7). Deactivation of the sadness response induced by films, music excerpts, and standardized imagery was associated with decreased respiratory activity, as indicated by a decrease in RR (63, 64). The change in RR can be affected by individual differences. Masaoka and Homma (62) showed that the respiratory response to mental stress and physical load is related to personality anxiety.

The FT serves as an indicator of the changes in blood flow due to vascular reactivity, reflecting the ANS response (12). It is primarily affected by sympathetic adrenergic vasoconstrictor nerves, and the activation of the sympathetic system results in vasoconstriction in the extremities, leading to lower extremity temperatures (65). The temperature showed a significant change under emotional stress. As the muscles become more tense, the blood vessels contract and the extremity temperature decreases. FT is decreased by stress and fear and increased by relaxation, boredom, and sleep (12). FT did not change during physical pain but significantly decreased during social pain, indicating that a sufficient amount of emotional stress is induced by social pain but not physical pain.

## 5 Limitations

In this study, we used a clip from a Korean drama to elicit social pain. It is possible that some participants have already watched the drama, thus being familiar with the content of the film clip, which could have influenced the study results. However, this study did not explore the effects of this familiarity on the participants’ responses. In future studies, we plan to gather information about participants’ prior knowledge of the stimulus to assess whether familiarity significantly impacts their responses. Additionally, while we chose a black-and-white color scheme (a black plus sign on a white background), it is important to acknowledge that color perception can affect an individual’s stress levels. Therefore, we must consider the potential impact of color perception on participants’ responses to stimuli.

The ANS responses to social pain were similar to those to sadness. According to Kriebig’s (7) review, previous studies that used films for sadness induction reported increased HR (64) and EDA (64, 66, 67) and decreased BVP, PTT, RR, and FT (64). For participants who cry in response to a sadness-inducing film clip, previous studies unanimously reported an increased HR, which was also associated with increased SCL and decreased BVP and FT (68). Contrastingly, sadness participants who did not cry while watching the film clip showed decreased HRV, represented by spectral respiratory sinus arrhythmia (66, 67) and decreased FT (68). In this study, social pain induced increased HR and SC features and decreased BVP and FT, similar to those observed in participants who crying in response to a sadness-inducing film clip. Simultaneously, the social pain used in this study led to decreased FT values, similar to those of sad participants who did not cry while watching a film clip. Because our stimulus was designed to induce social pain through the imminence of loss or loss of relationships, such as the death of the father, participants were likely to feel sadness. However, additional emotional assessment is needed to determine whether the stimulus used for social pain induces sadness. Social pain induced by the loss of relationships could also include threats, fear, or social distress owing to social rejection. Prior studies indicated that social pain such as social rejection evokes increases in HR and SCL (69, 70). Thus, diverse stimuli for inducing social pain are needed to examine social pain-specific ANS responses.

This study differentiated pain into two types—social and physical—and assumed that these two types were distinct. However, a wide range of factors related to pain stimuli and their responses are required. We identified ANS responses related to social pain using loss of relationship as psychological pain; however, psychological or emotional pain can arise from various causes when psychological needs (e.g., affiliation or achievement) are frustrated or the need to avoid harm, shame, or embarrassment occurs (71). Pain can be evoked by mixing different emotions, such as fear and sadness (72) and may be accompanied by other emotions [e.g., fear, sadness, anger, anxiety, and shame (27, 73)]. Owing to the diverse definitions of psychological pain and the various methods employed to induce pain, the evaluation of responses to such pain is complex. Physical pain could lead to different responses depending on the properties of the stimuli, such as heat, cold, pressure, and pricking. Further research is necessary to determine the specific types of pain stimuli that should be administered.

## 6 Conclusion

This study did not consider the participants’ familiarity with the stimulus content, the possibility of inducing emotions through the stimulus, and the various types of pain stimuli to verify pain-specific ANS responses. Nonetheless, we identified changes in ANS responses to social and physical pain stimuli and differences in ANS responses between the two pain types. Social pain induced increased HR and SC features, and decreased BVP, PTT, RR, and FT, suggesting a heterogeneous pattern of sympathetic–parasympathetic coactivation. Moreover, physical pain induced increased HRV features and SC features, decreased BVP and PTT, and resulted in no change in FT, indicating SAM activation and peripheral vasoconstriction, which is consistent with our previous study (12). These results suggest that changes in HR, HRV indices, RR, and FT can serve as significant markers for differentiating physiological responses to social and physical pain stimuli.

## Data availability statement

The datasets presented in this article are not readily available because of privacy restrictions. Requests to access the datasets should be directed to swbyun@inu.ac.kr.

## Ethics statement

The studies involving humans were approved by the Institutional Review Board of Chungnam National University. The studies were conducted in accordance with the local legislation and institutional requirements. The participants provided their written informed consent to participate in this study.

## Author contributions

E-HJ: Conceptualization, Data curation, Formal analysis, Methodology, Visualization¸ Writing – original draft, Writing – review & editing. Y-JE: Writing – original draft, Writing – review & editing, Conceptualization, Data curation, Formal analysis, Methodology, Visualization. DY: Funding acquisition, Writing – review & editing. J-HS: Writing – review & editing, Supervision. SB: Funding acquisition, Writing – original draft, Writing – review & editing.
